# Examining Alternatives to Painful Piglet Castration Within the Contexts of Markets and Stakeholders: A Comparison of Four EU Countries

**DOI:** 10.3390/ani11020486

**Published:** 2021-02-12

**Authors:** Li Lin-Schilstra, Paul T.M. Ingenbleek

**Affiliations:** 1 College of Economics and Management, Nanjing Agricultural University, Nanjing 210095, China; 2 Marketing and Consumer Behaviour Group, Wageningen University and Research, 6706KN Wageningen, The Netherlands; paul.ingenbleek@wur.nl

**Keywords:** animal welfare, pig castration, European union, multiple case studies

## Abstract

**Simple Summary:**

Painful castration of male piglets to avoid boar taint can potentially be replaced by three more ethical alternatives: using pain relief during and after castration, raising entire males, and administering vaccines. To ensure that pigs and pig products can be traded with the fewest barriers possible, the European Commission initially prefers the adoption of a single solution for the entire European Union. Each alternative is subject to its own advantages and disadvantages, however, and production chains in different member states disagree with regard to which alternative should be selected. This study examines the issue of castration in four different countries (the Netherlands, France, Slovenia, and Germany) against the specific background of their pork-production systems. The results indicate that, although the stakeholders in these countries are generally willing to switch from painful castration to one of the alternatives, the specific alternatives that they prefer are strongly dependent on the structure, scale, and cost and quality orientation of the production system. To improve animal welfare, policymakers should therefore consider allowing the coexistence of several different alternatives throughout Europe and developing policies for the most suitable alternatives for the pork-production systems in their own countries.

**Abstract:**

To avoid the occurrence of boar taint in pork, the castration of piglets without pain relief is a common practice in many European countries. The public has been calling for more animal-friendly alternatives, which include anesthesia/analgesia, immunocastration, and the raising of entire males. To prevent potential trade barriers, the European Commission was initially more in favour of a single method. To date, however, only six countries have passed laws banning castration, and the pig farmers in these countries have chosen different alternatives. To understand the reasons behind the continuing fragmentation, this study examines the issue of castration within the context of four national pork production systems: in the Netherlands, France, Slovenia, and Germany. Drawing on in-depth qualitative data, the study demonstrates that stakeholders are generally willing to abandon the practice of piglet castration without anesthesia/analgesia. Their preferences for alternatives are largely dependent on contextual factors, however, including the structure, scale, and cost and quality orientation of the production system. The results imply that, although a single solution for castration is unlikely to evolve amongst the diverse pork-production systems in Europe, a future without the painful castration of piglets is possible if alternatives are accepted to coexist.

## 1. Introduction

Each year in the European Union, about 90 million piglets undergo surgical castration (SC): a procedure in which the testicles are physically removed. The major purpose for the continuing practice of castration is to reduce the risk of boar taint, an odor that some customers find unpleasant, or even offensive when cooking and eating pork products. Sensitive consumers have described the smell as resembling urine, manure, mothballs, and sweat. Boar taint is caused by the accumulation of skatole and androsterone in the tissues of uncastrated males [1]. By castrating male piglets, producers intend to lower the level of skatole and androsterone. The procedure is nevertheless considered a poor practice from the perspective of animal welfare.

*...[Castration is] detrimental to the welfare of pigs, especially when carried out by incompetent and inexperienced persons. As a consequence, rules should be laid down to ensure better practices*

COUNCIL DIRECTIVE 2008/120/EC of 18 December 2008

In 2010, 33 stakeholders in the pork supply chain (including scientists, veterinarians and animal-welfare organizations) voluntarily signed an agreement: the European Declaration on alternatives to surgical castration of pigs. The agreement aimed to eliminate the surgical castration of pigs without pain relief by 2012, thereafter phasing out the surgical castration of pigs in all EU and European Free Trade Association (EFTA) countries by 2018. To date, neither of the goals have been achieved. Three alternatives are available to pork-production sectors: surgical castration (SC) using pain relief, immunocastration (IC), and the raising of entire males (EM). In the first method, castration is performed with the use of pain relief, primarily through the application of general or local anesthesia and/or analgesia. Anaesthetics and analgesics for piglets are not authorized in all EU countries and, in many countries, their use is restricted exclusively to veterinary surgeons. With the EM method, piglets are left uncastrated, and the slaughterhouses apply detection methods to identify tainted carcasses before moving the meat further along the production line. Immunocastration refers to the use of a commercial vaccine (Improvac^®^), produced by Zoetis Ltd. (formerly Pfizer Ltd.), to stop the production of male hormones (i.e., gonadotropin-releasing hormone; GnRH). If applied appropriately by farmers, the vaccine could largely reduce the incidence of boar taint. All three of these methods are currently in use within the EU. The advantages and disadvantages of the alternatives are listed in Table 1 [2,3].

The European Commission would like to arrive at a harmonized solution in order to facilitate strong economic ties within the common market. The process is highly complex, however, given that each of the three alternatives is subject to advantages and disadvantages that, for a variety of reasons, are likely to make them attractive (or unattractive) to particular groups of stakeholders, including breeders, farmers, processors, retailers, veterinarians, and NGOs. Moreover, the size and power of these stakeholders vary from one EU member state to another. After more than three decades of research and debates, stakeholders in the EU have yet to agree upon a harmonized approach to end the pain that piglets experience due to castration. Research from a socioeconomic perspective could help to clarify the barriers existing within the process.

To date, scientific attention has been devoted to validating the production effectiveness of each method in terms of economic benefits [4], meat quality [5], and animal welfare [6]. Several consumer studies have focused on consumer attitudes and sensory perceptions towards pork products with regard to the various alternatives [7,8]. Scientific research has nevertheless largely ignored the voices, opinions and positions of stakeholders with regard to piglet castration, despite the particular merits that such aspects could contribute to research on the issue.

The current study addresses this gap in the literature by examining the castration issue against the background of four EU member states, each with its own national pork-production system: the Netherlands, France, Slovenia, and Germany. The investigation focuses on four questions: (1) how the issue of castration has developed in each of the four countries; (2) how differences in the development of the issue can be traced to differences in the production and market structures of the countries; (3) how the structure of the pork-production system has influenced the decisions of stakeholders with regard to the issue of castration; (4) the directions in which stakeholders in the various countries are likely to move. By exploring these questions, we hope to inform the public debate and provide constructive input for animal-welfare policy within the context of the European common market.

## 2. Materials and Methods

Detailed stakeholder views about the structure of the pork production and market system are nuanced and complex, as are the ways in which society influences these systems and future developments. This study is therefore based on qualitative methods. More specifically, it consists of case study research, which allow in-depth, multifaceted exploration of complex issues in their real-life settings [9]. This methodology is particularly useful for clarifying and explaining why certain events happen. It allows for a critical, reflective perspective that considers the broader social and political environment that is likely to shape a given case [10]. In-depth case analysis can thus generate concrete, context-dependent knowledge for complex social problems, like the issue of piglet castration [11,12,13]. We define our cases at the country level, thereby allowing us to compare the issue across countries and against the background of each country’s systems for producing and marketing pork. According to the methodological directions developed by Yin [12], theories provide the starting point for investigating cases. We therefore build on existing literature to develop the concepts for the case study and to select representative countries for the cases.

### 2.1. Case Study Concepts

Given that piglet castration can be seen as a social issue that evolves differently in different countries, our study is guided by three case-study concepts: social issue management, the structure of production and market systems, and stakeholder engagement and dialogue.

**Social issue management.** A social issue is defined broadly as a problem that is recognized by the public as being disruptive or damaging to a group or to society in general. In the case of piglet castration, public recognition of the issue varies amongst European countries. In most countries in Eastern Europe, piglet castration is not yet considered a social issue [13]. In these countries, castration without pain relief of any kind continues to be standard practice and, to date, the issue has failed to receive widespread recognition, and there has been little or no pressure from society to change the practice. This suggests that the issue of castration remains at a stage of insignificance. In other countries (e.g., Denmark and the Netherlands), the issue has drawn public attention to such an extent that policymakers have passed laws to reduce the pain associated with castration by banning the practice of castrating young male pigs without anesthetics (see Table 2). To examine and compare the different stages of issue development, we draw upon the literature on social issue management, which distinguishes three predictable stages of issue development: a period of insignificance; a period of increased attention, awareness and conflict; a period in which new solutions concerning the issue become formalized within the society [14]. This process is shaped by strong social movements and activists, powerful stakeholders or entrepreneurs entering the field with new technology [14,15,16].

The stage of social issue development also influences stakeholder behavior [14,18]. The extent to which a social issue has reached consensus at the societal level affects the ways in which stakeholders frame an issue and whether they will or will not engage in it [19,20]. Each of the different stages of issue development is therefore accompanied by different risks and opportunities for the actors in the production chain. In countries like the Netherlands and Denmark, where the public is aware of and holds a generally critical attitude towards painful piglet castration, changing the practice offers an opportunity for the sector to strengthen its relationship with retailers that are the gatekeeper for consumers. Likewise, failure to respond to the issue is associated with a risk of bad press and consumer boycotts. In contrast, in Eastern Europe, where the media devote significantly less attention to the issue, farmers are logically also more hesitant to change. Building on these observations, we asked interviewees to identify the risks and opportunities associated with the issue. The following are several examples of the questions. “What are the greatest challenges that the issue of piglet castration poses for the pork industry in [country] in general?” “Compared to the situation of the pork industry five years ago, how has the issue of castration influenced your business?”

**The structure of production and market system of the pork industry.** Whether and in what way actors in the pork production chain will work towards a change will also depend on whether their decisions would disrupt the existing consonance within the existing production system [20,21]. In other words, producers refer to the macro-level production/market environment for decision-making [21]. More specifically, they consider whether the macro environment is suitable or ready for a change in business practices. Aspects of key concern include multiple factors, including the size of production units at various levels of primary production, the consolidation of slaughterhouses and processing companies, concentration at the level of the retailer [22], the presence of special and regional products, and the heterogeneity of consumer demands [23].

**Stakeholder engagement and debate.** Arriving at a single solution to the issue of piglet castration inevitably requires an agreement between various stakeholders, including farmers, veterinarians, slaughterhouses, and retailer organizations. For this reason, we also consider the literature of stakeholder engagement and debate. Whether a social issue will advance to actors within companies will depend at least in part, on the way issues are communicated amongst stakeholders. Communication activities involve more than the one-way dissemination of information, as producers are embedded within the network of the production chain [24]. With regard to the issue of piglet castration, production actors interact with each other to exchange information, discuss opinions and expectations, and, to some extent, influence each other. Such engagement takes the form of stakeholder dialogue or, if multiple stakeholders are participating, a debate. A debate that meets a number of necessary conditions (e.g., respect, equal opportunity, transparency, trust, and mutual interests) is likely to progress towards a solution [25]. Stakeholder interactions can affect the direction of issue development, the interests of groups involved, and public policy [26]. The goal of stakeholder dialogue is to develop new common standards and reach some type of agreement [27] that can provide stakeholders with guidance about what action is acceptable and desirable.

In this study, we investigate the positions of stakeholders with regard to the issue of piglet castration. To this end, we explore the bases on which stakeholders claim that their positions are legitimate and the ways in which they seek to exercise influence within the production chain concerning their standpoints. The following are amongst the questions posed to our interviewees. “Given that three methods are available, how do you evaluate the economic benefits of each new method for your organization?” “You suggest that the method of [castration with pain relief/raising entire males/immunocastration] is better than the other two. Is your organization applying it? If so, do you encounter any difficulties? If not, what are the major obstacles that are preventing your organization from using your preferred method?” Questions intended to explore the stakeholder debate include, “What were you hoping to accomplish in the stakeholder meetings or discussions in which you participated?”

### 2.2. Selection of Four Case Countries

Guided by the case study concepts discussed above, we developed criteria for selecting representative countries. From the perspective of social issue management, one important criterion involves variation with regard to the stages of issue development. Several Western/Northern European countries are ahead of the rest of Europe in the process of abandoning castration without the use of pain relief (Table 2) and applying alternatives to castration. Criteria related to the concept of structure include characteristics of the pork sector in terms of production structure (extensive vs. intensive) and in terms of market structure (export-oriented vs. import-oriented). With regard to stakeholder debate and engagement, we selected countries in which nongovernmental organizations (NGOs) are active in launching campaigns, which usually lead to stakeholder discussions. Several nation-wide campaigns have been held in France and Germany in recent years. Variation amongst the selected countries in terms of these criteria is important, in order to allow multicountry comparisons that can identify similarities and differences, in addition to relating them to the positions of stakeholders.

Pork chains in European countries differ in terms of the products they produce and the markets on which they focus. Within this context of diversity, our research addresses the issue of piglet castration as one of many animal-welfare issues within the industry that call for resolution. The Netherlands, France, Slovenia, and Germany met the criteria for comparative case studies. The Netherlands was one of the earliest countries that recognize the issue of castration and, accordingly, it has been making efforts to advance the issue [18,28]. Germany was selected because of its decisive role in the future development of the issue, given its economic and political power within the EU. France was selected because of several nationwide campaigns that have been held in recent years, during which increasing numbers of pig producers have become involved in the discussion concerning the issue. France also has a typical market, in which consumers attach great importance to high-quality meat. The Slovenian market is largely silent on the issue, which makes it a representative case for Eastern Europe [28]. The choice of the four countries was confirmed in a discussion meeting with a group of 10 senior experts from the SuSI (Sustainability in pork production with immunocastration) project [29].

### 2.3. Data Collection

Semistructured interviews were conducted in order to optimize the collection of information for use in answering questions concerning how the structure of the pork-production system may has influenced the decisions of stakeholders with regard to the issue of castration and the directions in which stakeholders in each country are likely to move. Initial contact with the experts was made through partners in the project consortium. These contacts were expanded using the snowball technique [30]. In-depth interviews make it possible to learn about motives, practices, and interactions based on the perceptions and perspectives of other stakeholders. In all, 39 experts/representatives of stakeholders agreed to participate in the study: the Netherlands (7), France (8), Slovenia (10), and Germany (9). The remaining five interviewees were working for organizations/institutions outside these countries.

The interviews were conducted face-to-face whenever possible within the scheduling of field visits and language constraints. Interviews with participants from the Netherlands, France, and Slovenia were conducted face-to-face in English by the first author. The nine interviews with German stakeholders were conducted by telephone by a trained research assistant who speaks German as a first language. The five interviews with stakeholders who were not from the selected countries were conducted by Skype, due to the inconvenience of travel. These respondents were asked to comment on the challenges that the issue poses within the European context. Their comments were incorporated into the result section in this study. Each interview lasted approximately 1.5–2 h. They were consulted based on their international expertise on piglet castration, which could provide additional information concerning all four case countries. All interview recordings were transcribed for analysis.

The semistructured interview format allowed for informal conversation on topics relating to the interviewees’ operational practices and interactions with other stakeholders, as well as with regard to the ways in which participants perceived that they and their organizations were coping with the issue of piglet castration. Derived from the main concepts, three main topics were selected in order to be addressed during the interviews. First, in relation to the concept of social issue management, the interviewees were asked what they regarded as the most important challenge posed by the castration issue. The following is an example of questions in this regard: “From a general perspective, what are the greatest challenges that the issue of piglet castration poses to the industry in [the Netherlands/France/Slovenia/Germany]?” The interviews continued with questions concerning which alternatives the respondents preferred and why. The following is an example of questions in this regard: “Each alternative method has a downside. What are the disadvantages of the various methods for your organization?” The second set of questions related to the concept of stakeholder engagement, aiming to identify disturbances in the supply chain. Interviewees were asked to recall disagreements that they had encountered with other stakeholders in the course of the stakeholder debate. The following is an example of a question in this regard, “Have you observed that other stakeholders are interested in other alternative methods?” The interview continued with questions concerning the adjustments that are needed in the supply chain. The following is an example of questions in this regard, “Assuming that the supply chain is going to adopt the method that you prefer, which adjustments will need to be made within the supply chain?” The final set of questions was intended to explore the predictions/visions of the respondents concerning how the issue should be and will continue to be approached in the future, and how their business strategies should be adjusted accordingly. An interview guide was used in order to provide a framework for discussion and consistency in collecting data from each interviewee (Appendix A).

### 2.4. Data Analysis

The analysis of the data involved a process of open and selective coding [31]. The software package Atlas.ti 8 was used to support the coding of transcripts. As the coder was also the interviewer for the interviews conducted in the Netherlands, France, and Slovenia, it was only necessary to read through the interview transcripts from Germany to develop an understanding of the discourse in in that country. After having read these transcripts, the coder used the method of open coding. Initial coding focused on the interviewees’ (1) perceptions about the challenges posed by the castration issue and their preferences for alternatives, based on their reasoning concerning advantages and disadvantages; (2) perceptions of working in the supply chain; (3) positions/visions for the future. These aspects were treated as central categories for analysis in each country.

### 2.5. Compliance with Ethical Standards

The study was supported by the Netherlands Organization for Scientific Research as part of the ERA-Net Cofund SusAn (grantnr 696231) through a virtual common pot model with EU top-up. Both authors, Dr. Paul Ingenbleek and Dr. Li Lin-Schilstra declare that they have no conflicts of interest.

All procedures performed in studies involving human participants were in accordance with the ethical standards of the institutional and/or national research committee and with the 1964 Helsinki Declaration and its later amendments or comparable ethical standards. Informed consent was obtained from all individual participants involved in the study.

## 3. Results

In this section, we answer the research questions by reporting how four countries differ in terms of (a) the market position (export/import) within the European pig sector; (b) the structure of the pork production and marketing system; (c) the extent to which the castration of male piglets is a social issue. The percentage of pigs raised as entire males, immunocastrates, and castrates in four countries is displayed in Table 3. Following the description of each country’s production/market system, we present the ways in which the decisions of stakeholders are influenced by the system and the issue. Interviewees discussed the challenges posed by the castration issue and their engagement in the stakeholder debate, using this reasoning to state their expectations concerning the future of the issue development. It is important to note that some stakeholders referred to the analysis of advantages and disadvantages in their position statements. As shown in Table 1, none of these alternatives are perfect. The positions of the respondents regarding the castration issue are presented in Table 4.

### 3.1. The Netherlands

#### 3.1.1. The Structure of the Production/Market System and the Development of the Issue

Pig production is an important economic activity in the Netherlands. In terms of export volume, the Dutch pig sector was the fifth largest in the world in 2019 (after Spain, United States, Germany, and Denmark). In that year, the Dutch pork industry exported more than USD 2.7 billion, accounting for about 72% of its production [34]. According to estimates in 2019, Germany remained the top destination for pork exports, accounting for 25% of all sales [35]. Imports and exports between the Netherlands and Germany are subject to the mutual conditions established by quality-control systems in the Netherlands (IKB, Integrale Keten Beheersing) and in Germany (QS, Qualitäts Sicherung).

For a country that is heavily dependent on exports, the realization of a low-cost production is important. Overall, the Dutch pork system is strongly oriented towards cost-efficiency [35]. Intensive production systems are dominant in the Netherlands. More than 90% of the output is produced by farms with herd sizes exceeding 400 pigs. The largest slaughterhouse, VION, accounts for more than 70% of the market. Farmer cooperatives play an important role in the pork sector—VION is fully owned by farmer cooperatives—although they are not involved in its day-to-day business operations. With regard to retailer concentration in the market, the five largest retailers hold market shares of up to 80% [23,36]. Special regional products account for a small amount of market share in this country. Any changes in the industry are likely to involve a preference for options that optimize cost-efficiency with outstanding production quality.

The Netherlands was one of the first countries to start looking for alternatives to pig castration due to ethical reasons. In 2000, a working group consisting of stakeholders from NGOs, veterinarians, and the industry was established to investigate the issue. The working group searched and validated all existing alternatives. Based on the results of their investigation, the stakeholders adopted the “Noordwijk Declaration” in 2007, in which they agreed to abandon the practice of castration without pain relief. Any pigs that are castrated receive general anesthesia involving the inhalation of carbon dioxide, which is commonly used for pre-slaughter stunning though it produces strong aversion (irritation and asphyxia) in pigs before they lose consciousness, The production of entire males increased rapidly after the adoption of the declaration. A study conducted in 2016 reported that the percentage of pigs produced as entire males had reached as high as 60%. At present, all large Dutch retailers sell fresh pork that has at least one star in the Beter Leven (Better Life) certification system, which explicitly specifies uncastrated pigs as a minimum requirement.

The Netherlands is one of several countries that have approached piglet castration as an animal-welfare issue and have made early efforts to resolve it. The 2007 Noordwijk Declaration, which was intended to improve animal welfare, states the ambition to ban the castration of male piglets by 2015. After more than 20 years of development, the issue of castration has been relatively settled with the solution of raising entire males. As confirmed by an active farmer representative:

*We did research to look for all possible solutions. We agreed that raising entire males was the best option for us since the Noordwijk Declaration. (NL-FM)*

Experts identified several reasons that had contributed to the choice for the entire-male solution. First, national traditions influence the ways in which business sectors interact with social pressure groups and how they perceive each other. Interviewees frequently referred to the polder model (which refers to a Dutch tradition of collaboration) both within the chain and between the for-profit and not-for-profit sectors [37]. The way in which the Dutch pork industry dealt with the castration issue followed this model as well. As argued by a manager from the leading slaughterhouse:

*In the Netherlands, we have a stronger culture of discussing matters with each other than is the case in Germany. We call this the “polder model”. (NL-SH)*

Such an attitude of collaboration and open dialogue allows stakeholders to consider the advantages and disadvantages of each method from the perspective of the overall chain. As articulated by the farmer representative:

*I really think that the whole chain should see it as a complete practice. So, what kind of quality is desired? For example, consider the boar taint problem. People say that the odor is from boars, but it’s not. It’s because of the whole chain operation—from breeding, animal feeding and so on. When you raise boars, you have to have different feeding plans. So, I think those chains have to join forces in order to achieve a good result of tasty pork. (NL-FM)*

Another important aspect has to do with the influence of animal interest groups within the industry. In the Netherlands, animal interest groups enjoy a favorable reputation in society, due to their professional efforts to improve animal welfare in general. Some of these groups are specialized in exerting pressure on the chain and experienced in collaborating with industry and retailers [38]. The largest animal interest group, the Dutch Society for the Protection of Animals (DSPA), offers an example in the case of pig castration. From the time that the issue emerged in 1997/1998, DSPA has been involved with the issue and has taken a leading role in promoting the process of collaboration. Government-level intervention is limited in the process of chain collaboration. The DSPA introduced the criteria for piglet castration into the Beter Leven certification system (which usually assigns three stars for organic products and one star for products that are slightly above legal levels). It is important to note that the DSPA was not the sole party to determine the criteria. Stakeholders within the industry contributed as well, thus possibly segmenting the market in order to achieve both ethical and economic benefits.

Animal interest groups are convinced that raising piglets as entire males has the advantage of avoiding castration pain and maintaining the integrity of the animals. When asked about the organization’s opinion on the method of immunocastration, the DSPA stressed that the method is another way of altering animals for human needs. If there is an alternative that can preserve the intrinsic value of an animal (as is the case with the entire-male method), the industry should work to adopt it. As argued by an expert from DSPA:

*We are not in favor of immunocastration, which still involves altering animals to fit human needs. From an ethical point of view, we are opposed to it. Moreover, it poses a major interference in animal’s sexuality, and it’s not necessary in the Netherlands, as the problem can already be solved by raising entire males. (NL-NGO)*

A third aspect that can promote change within the production chain is cost-efficiency. By converting to the option of raising entire males, the production system stands to benefit from a favorable rate of feed conversion. The major difficulty involved in implementing this production method, however, is that it requires a fundamental change in the chain—from farmers to slaughterhouses/processors and, ultimately, to retailers. At the farm level, farmers must invest in farm management by raising the level of hygiene and handling more aggressive pigs in order to reduce the incidence of boar taint at the farm level. Further along the chain, slaughterhouses/processors invest in detecting tainted meat and making distinctions for products of differing requirements. For example, carcasses that have tested positive for boar taint can be used for processed meat products. As explained by a manager from a leading slaughterhouse:

*So, farmers need to adapt to managing boars. Management will have to adjust a little bit, but not much. We have implemented the detection of boars by smelling, and we are doing this already. We process them separately. (NL-SH)*

As the ultimate gatekeepers for consumers, retailers must jointly agree to a higher standard of animal welfare. In the Netherlands, major retailers have agreed that meat from entire males meets the standards of both quality and animal welfare. With support from DSPA, the industry added the criteria of entire males to the Beter Leven certification system. This raised the standard of animal welfare for stakeholders and created consensus understanding with regard to the preferred solution. The certification scheme also defines the minimum level of animal-friendly products for Dutch consumers. Accordingly, even consumers who only buy inexpensive products are contributing to a higher standard of animal welfare. The criteria for the Beter Leven labels are published online, and interested consumers can easily find the information. With regard to the industry’s decision to adopt the method of raising entire males, a manager from a leading supermarket noted that:

*The need for an alternative is much lower than it was 10 years ago. (NL-RT)*

#### 3.1.2. Stakeholder Engagement

The industry’s decision to adopt the entire-male solution reduced the challenge posed by the issue of castration in the Dutch market. According to the interviewees, however, the main challenge posed by the issue now has to do with uncertainties in export markets, especially Germany, which holds extensive political and economic power in the EU, with Germans holding more key positions in the European Commission than any other European country [39]. Whether and how Germany will implement legislation that bans piglet castration is expected to have a significant impact on the pig sector in Germany, as well as throughout the EU market, as this influential player will send signals to all other European countries with regard to resolving the issue. As Germany has postponed the effectuation of the ban on castration without pain relief by two more years (until 2021), however, there is considerable uncertainty in the German sector with regard to the actions that will be needed in order to prepare for the implementation of the castration ban. As pointed out by an independent researcher:

*Developments in Germany pose the greatest challenge to the Dutch pork industry. Their initial legislation banning castration has been postponed for two years. It is not entirely certain that it will actually be effectuated. (NL-ST)*

As the Dutch supply chain for pork is export-oriented, the extent to which the export markets will accept pork from entire males is likely to bear a major influence on the competitiveness of the Dutch pig sector. Therefore, the experts emphasized that establishing a level playing field is needed for the mutual acceptance in the European market. Together with the level playing field, production actors should adopt a “market-driven” concept. To facilitate trading relations with Germany, efforts have been made to establish frameworks or protocols for human nose testing—a notably harmonized effort between quality-assurance companies in Germany (QS) and the Netherlands (IKB). The provisions of the protocol established by QS and IKB include a requirement that it be followed by all slaughterhouses that are engaged in processing entire males. As emphasized by the farmer representative:

*We are mostly talking to QS, and we have an agreement that they will accept our quality system and our way of working. (NL-FM)*

Another challenge involves the spreading of misinformation on the international market. Almost all of the Dutch interviewees expressed either direct or indirect concerns about the false information that is circulating throughout the industry. Such information can be classified according to two aspects. The first aspect concerns the technical features of each alternative. For example, an experienced scientist provided the following indirect explanation:

*What I see in the research community is that many scientists are dependent on funding. In many cases, the one who is paying for the funding decides which research is to be done. The point is not that the research is not conducted appropriately, but that it is biased towards a certain position. My null hypothesis is that, if large farmers could raise as much money for research as large companies can, the research project portfolio would change a lot. (NL-ST).*

A manager from a French cooperative used the following example to express a similar concern:

[There are people who say that] *it is impossible [to raise and sell] pigs older than six months because they would smell. That’s false. (FR-CO)*

The other aspect is related to the acceptance of the international market, especially in Asia. Concerns for the existence of misinformation were also shared by the experts who were interviewed in France, Germany, and Slovenia. According to a representative from the largest cooperative in France:

*It makes me nervous to listen to these mistakes. People say, they seem to be certain in saying that the Chinese population doesn’t eat entire-male pork and things like that. It makes me nervous to hear that, because I know it’s false. (FR-CO)*

#### 3.1.3. Perceptions with Regard to Future Changes

Most producers in the Netherlands were convinced that the entire-male solution offered the best and ideal alternative to castrating piglets from the perspective of both ethics and economics. The issue is relatively settled in the Netherlands. For the purpose of equal trading, an agreement has been made with German producers that the supply of conventional meat in the Netherlands, including imports, is to be sourced from entire males. Imports from Germany must comply with the Dutch IKB regulations. To stay abreast of developments in the international market, key players hold regular meetings to exchange information or organize seminars to show how they manage boar taint by raising entire males. As argued by a farmer representative:

*We are not going all over Europe to lobby for our products. What we do is, for instance, French supermarkets have questions about raising entire males. We go there and tell the story, what our aim is and what we are doing. (NL-FM).*

With regard to the uncertain situation in Germany, the interviewees apparently did not feel a sense of urgency to adjust their current business. At the same time, however, they did keep themselves updated on the development of the issue in Germany, as it would not be technically difficult or costly to adopt the other two alternatives (i.e., castration with anesthesia or immunocastration). If the international market were to demand such products, Dutch producers are flexible enough to adapt to the requirements of the foreign market. As noted by the manager from the leading slaughterhouse:

*We are quite flexible. (NL-SH)*

### 3.2. France

#### 3.2.1. The Structure of the Production/Market System and the Development of the Issue

The French pork industry is less reliant on exports than is the case for the pork sector in the Netherlands. France exports 25% of its production. The main destinations for this meat are countries in the European Union: Italy, Spain, Belgium, the United Kingdom, and Germany, along with other countries (e.g., China and the Philippines) [40]. In the meantime, up to 25% of the pork consumed in France is imported [40].

Pig farming is somewhat less intense in France than it is in the Netherlands. Small farms (less than 10 pigs) and medium-sized farms (10–400 pigs) account for about 10–25% of the country’s production [41]. More than 75% of France’s swine production is concentrated in livestock farms located in the French Great West, and especially in the regions of Brittany and Pays de la Loire [42].

Since 2016, the castration issue has been gradually attracting public attention in France. In that year, the French animal-welfare organization WELFARM launched several tough anticastration campaigns. A poster campaign known as “Couic”, which mimicked the sound of cutting testicles, appeared in various metro stations and social media. Since that campaign, public pressure has been increasing. By the end of 2019, France announced a ban on piglet castration without anesthesia, which is due to take effect at the end of 2021. When asked about the motivation of holding the campaign, a campaigner in WELFARM responded:

*The main objective is to convince politicians to pass a law on pig farms that will impose a total ban on piglet castration for the reason of pig welfare. So, we are trying to collect public opinions on the subject. But we are also pressuring retailers, breeders and pharmaceutical companies to cooperate with us. Veterinarians, slaughterhouses and all the main stakeholders should all be working in the same direction. (F-NGO)*

One important characteristic of French meat production, including pork production, is an emphasis on the quality of meat [43]. The country is known for having some of the best food in the world, not only in terms of recipes, but also with regard to the sourcing of ingredients. In the market, French products are subject to many high requirements concerning the quality of meat products. Pork producers can be certified with a variety of quality labels. For example, the famous Label Rouge, which is quite familiar to French consumers, certifies specific product characteristics and demonstrates that products are of higher quality than standard products [44]. The ideology of authentic food affects the ways in which French pig producers perceive the balance between meat quality and animal welfare. A representative from the farmer association stressed the importance of considering the quality requirements of consumers in their business operations:

*The first concern for consumers in France is quality. The second should be the welfare. They will never accept greater welfare if the quality is not good enough. (F-FM)*

The attention to meat quality guides the ways in which some French producers evaluate alternatives to castration. Boar taint is only one of several factors that are considered when assessing the quality of pork. Other important factors include the ratio of fat to lean meat, tenderness, and fat quality. All of these factors are taken into account when assessing various production methods. In addition, there is a large market for regional and specialty processed products, as well as the market for bulk fresh meat in France (Rakotonandraina and Sauvee, 2008). For these products, meat from entire males (lower ratio of fat to lean meat, less tenderness, and lower fat quality) is believed to be less appealing to consumers. As emphasized by the representative from the farmers’ association:

*The quantity of fat is not nearly the same [between entire males and immunocastrates]. And for a few products in France, we need a little bit more fat. For example, dry ham. For this type of product, it’s the reason why some farmers cannot and would never want to produce entire males. The quality is just not good enough to produce this type of product. (F-FM)*

Regardless of these views, the largest French pork-production group (Cooperl), which accounts for 20% of all pig production in France, has not been practicing castration since 2013. Cooperl is a cooperative that integrates feed producers, farmers, slaughterhouses, and processors. Farmers who are not integrated into the Cooperl group still perform castration. According to one survey, the percentage of farms applying anesthesia or analgesia during castration is believed to be very low [17], as is the number of farms using immunocastration (according to an estimate by the manager from Zoetis, which manufactures vaccines for immunocastration. The company was a subsidiary of Pfizer, the world’s largest drug maker. With Pfizer’s spinoff of its 83% interest in the firm, however, it is now a completely independent company).

#### 3.2.2. Stakeholder Engagement

In France, only Cooperl has transitioned to producing meat from entire males. It provides an example of successful chain transition supported by effective coordination. Cooperl integrates feed producers, farmers, slaughterhouses, and processors. The transition was inspired by the example of the Dutch transition to entire males and its early commitment to animal welfare. In 2009/2010, Cooperl launched a trial study exploring the feasibility of both immunocastration and the entire-male method. The evaluation, which involved calculating the collective costs and benefits to the production chain, concluded that transition to entire males would yield more benefits to Cooperl as a cooperative. To support the transition to the production of entire males, comprehensive protocols have been introduced to guide farm management (e.g., the change of diet, on-farm management for aggressive behavior) and the detection of tainted carcasses in slaughter lines. A bonus-penalty system has been introduced to encourage farmers to adopt more preventive practices in raising entire males. As noted by one Cooperl manager:

*With our farmers, we share technical and economic data. We have implemented a simulator. The factors that have the greatest impact include the staffing situation, regarding food conversion, lean percentage, slaughter age, weight, etc. And then, with our experience in genetics, we can make some predictions, as farmers can potentially add value in terms of food conversion and slaughter weight. But they might also detract value due to carcasses with boar taint carcass. We pay less for those carcasses. The system assigns value to farms. (F-CO)*

It is almost impossible for other farmers to copy the practices of Cooperl. Many other farms and slaughterhouses are independent of each other. They are not as integrated as Cooperl. It would be difficult for these independent farms and independent slaughterhouses to change to any alternative. Without a mechanism of coordination (e.g., a coordination platform or following a tradition like the “polder model”), independent farmers and independent slaughterhouses have fewer interests in converting to producing, processing and selling meat sourced from entire males or immunocastrates. As farmers receive the most criticism for the unethical castration of pigs, the farmer representative was convinced that farmers should assume the major responsibility for finding the detection method for slaughterhouses:

*We talked with the slaughterhouses to share the cost of researching. But they are not the leader in this issue. It’s up to the farmer to do the research. When the IFIP (French pork and pig institute) researches it, the farmers are paying for them, not the slaughterhouses. If the farms find a good detection solution, we can start our discussion with the slaughterhouses about breeding more entire males. (F-FM)*

The animal interest group, WELFARM, identified this missing link between farmers and slaughterhouses. After six years of campaigning and communicating activity with regard to the castration issue, the organization is now in contact with several farmers, slaughterhouses and retailers. WELFARM is able to assume a partial role as a coordinator, helping a few farmers to come into contact with independent slaughterhouses that were willing to accept entire males. The leader of the castration campaign explained how they played their coordinating role with the industry:

*We speak with retailers. We say, well, these breeders want to move [to entire males], but they are waiting for your confirmation. You as a retailer said that you want to buy entire males…In this way, I tried to bring breeders and retailers into contact with each other. (F-NGO)*

In France, the industry does not seem to be very interested in the alternative of immunocastration. To date, only a few local production chains are applying this method. According to an estimate from a manager in Zoetis, the market is less than 1%. The company is currently less actively involved in the issue than it was 10 years ago:

*We get some Improvac users. Most of them started six or seven years ago, and they’re still using it now. But all of them are selling their products directly [to local consumers]. They are very short chains. Now, only a small group of farmers are using Improvac. But the good thing is that people* [who are using Improvac] *like the product. (F-PH)*

From the perspective of animal welfare, Zoetis also lacks the support of the animal interest group. The WELFARM group has a clear preference for the entire-male solution over immunocastration. The campaigner stated two reasons. The first reason was that:

*Zoetis is unfortunately the only stakeholder in France who owns immunovaccines. It’s kind of complicated for us. We are not the Zoetis commercial. (F-NGO)*

The second reason was that:

*We think that raising entire males is the best solution, as it does not involve any manipulation on animals. We understand that some breeders [who produce for dry hams or organic farmers] can’t use entire males. Immunocastration could be a good solution for them. We think that it would be better to have both of these solutions. We just ban castration. Then we say to the breeders, “You can deal with both of these two alternatives”. (F-NGO)*

#### 3.2.3. Perceptions with Regard to Future Changes

With a common understanding about the importance of meat quality, farmers and researchers share a consensus understanding that the major challenge posed by the castration issue is the availability of an automatic and reliable detecting machine that can ensure 100% accuracy for tainted carcasses. As argued by an experienced scientist:

*The first challenge is to be able to assure the pork industry that there is no boar taint. The quality can be defended. The priority is to work on detection at the slaughterhouse. (F-ST^1^ )*

Cooperl confirmed that they will continue on the path of entire-male production, as the risk of boar taint has been greatly reduced by the joint efforts of farmers, slaughterhouses and processors in the integrated chain. This part of the entire-male production is likely to be stable. When asked about the future of the issue, experts estimated that the percentage of entire males in France will increase somewhat, but that it would be almost impossible to phase out castration completely, due to high quality requirements of consumers of special and regional products. The representative from a farmer association insisted that castration with pain relief should be allowed in France:

*Perhaps in the future, we can have more products from entire males. But the quality from entire males and immunocastrates is not good enough to produce dry ham. That is the reason why we would like for the farmers to have access to anesthesia. There are some farmers—in fact, part of the industry—who will never want to have entire males. (F-FM)*

With regard to the influence of Germany on France, most experts were convinced that the possibility that Germany would postpone the implementation of the castration ban would not have a large impact on the French pork industry. This is because the French pork sector is not highly reliant on exporting to Germany.

### 3.3. Slovenia

#### 3.3.1. The structure of the production/market system and the development of the issue

In terms of the market position in the EU, Slovenia is a net importer of pork. The self-sufficiency rate for pork in Slovenia is around 20–30%. The major importers are Austria, Hungary, and Spain, which together account for almost 70% of Slovenian total imports. Compared to international rivals, Slovenian producers are on a smaller scale for both the production and the slaughtering and processing of pigs [45].

Three distinguished production systems exist within the Slovenian pork industry [46]. Farms of over 400 pigs account for about 30% of all domestic production. Two large, vertically integrated pig enterprises—Farme Ihan and Panvita—are the main large-sized producers. Roughly 40% of all farms in Slovenia are medium-sized family farms, which keep 10–400 pigs. Small units of less than 10 pigs usually focus on preserving a particular character associated with traditional ways of farming, producing products labeled as “traditional”. In addition to selling pigs to local abattoirs (butchers/meat processors), they often practice home processing for the direct sale of products to consumers, or for family consumption. Such family farms are not integrated into any system. Pigs are slaughtered at higher age and weight and sold in a short supply chain. These small farms are important to the Slovenian market, representing approximately 30% of all domestic production [40]. Compared to the situations in the Netherlands and France, the overall production structure in Slovenia is more extensive. The small scale and scattered structure of Slovenian agriculture make it less suitable for mass production, which is aimed at minimizing the costs of production [45].

Slovenia has not yet implemented any laws banning the practice of piglet castration without pain relief. The most relevant political support consists of a special “improved welfare” regulation, in which farmers can apply for subsidies for the usage of anesthesia during castration. Only veterinarians have access to anesthetics. The cost of inviting veterinarians on-site to apply anesthesia is usually too high for the majority of small farms. In most cases, piglets are castrated without anesthesia. Some pigs are castrated at older ages, such that they endure severe pain. One 2018 report concludes that there is not really a sense of urgency for the pig sector in Slovenia to work towards ending surgical castration [28]. The castration issue also does not attract much attention from the media or from animal groups, and public attention to the issue is low.

#### 3.3.2. Stakeholder Engagement

Retailers have yet to exert pressure on the supply chain. The pig sector in Slovenia, including governmental institutions, does not feel a sense of urgency to work towards ending surgical castration. The level of stakeholder engagement about this issue is very low. Two integrated cooperatives—Farme Ihan and Panvita—conducted trials with immunocastration in 2009/2010, when the use of vaccines was approved in the EU. Cooperating with the Agricultural Institute of Slovenia (KIS), the two enterprises launched experiments with their farmers. The research was motivated by curiosity about the immunocastration method. Following a round of experiments, two enterprises concluded that the application of immunocastration in their farms was not profitable enough. According to a manager from Farme Ihan:

*We don’t consider immunocastration. The cost is too high. We now have a low margin because of competition from other countries. The price of their meat is very low. We do not have an advantage over these competitors. (SI-CO^1^)*

In the absence of pressure and sufficient financial potential for immunocastrates, producers in Slovenia have not felt any urgency to change their current practices. The major concern for Farme Ihan and Panvita is to maintain their competitiveness in the Slovenian market, rather than working towards a solution for an animal-welfare issue. Competition emerges from domestic family farms and international players. Another manager from Farme Ihan gave details about these two sources of competition:

*First, we have some kind of problems with small farms—they have only two or three pigs on the farm, because they want to have their own products. People from the village buy these products directly from these farmers. The farms are not traceable, but the number of small farms is expected to be relatively large. Second, we also face competition from other countries. Governments in these countries provide subsidies to their producers, but we don’t have them. The difference between the production cost in Slovenia and in other countries is between EUR 22 and EUR 26 per pig. Some Slovenian slaughterhouses process this imported meat. the low prices are advantageous to them. (SI-CO^2^)*

Both of the cooperatives maintain a strong focus on reducing costs in order to maintain their competitiveness in the Slovenian market. Additionally, the management boards of Farme Ihan and Panvita were convinced that promoting “traditional” meat was the right and only tactic for distinguishing Slovenian meat from imported meat. This is because some Slovenian consumers are willing to pay a higher price for traditional meat. As confirmed by a manager from a breeding company and a slaughterhouse:

*Traditional is very important. If consumers have the perception that the product is traditional Slovenian and non-industrial, they will be willing to pay a higher price. (SI-BR)*

The cooperatives nevertheless fear that the use of immunocastration would damage the image of “traditional” meat in the market. With regard to the method of entire-male production, they fear that such a shift would require costly investments in order to change the current manner of processing, thereby reducing their margins on the Slovenian market. Surgical castration with anesthesia is currently regarded as the most suitable solution by the two integrated chains. Although the application of anesthesia does increase the cost of castration somewhat, the practice does not disturb the procedures for farm management and processing, nor does it affect the quality of meat products.

Small family farms, which account for a substantial part of Slovenian domestic production, have little involvement in the castration issue, with the exception of reacting negatively to the publication of the trial experiment of the cooperatives in 2010. The KIS research institute, which published the paper, came under attack by these small family farms:

*We had some results, and we published them in a national journal in order to transfer some new knowledge. We intended to present what was taking place in Europe with regard to the issue and to raise awareness that this is something that will become an issue in pig production. We were then attacked, especially by the small farmers, who questioned our motivation, as if we were promoting immunocastration—that we were promoting some changes, as if we were responsible for what is happening, that we were forcing them to introduce the changes. (SI-ST)*

Since that time, KIS has decided to continue working with international research institutes, but to reduce the publication of its work at the national level. Researchers were convinced that it is difficult to communicate with family farmers, for the aforementioned reason of mistrust. The institute also noted that it did not receive sufficient support from the government:

*The chamber of agriculture has advisories, and they only work with family farms. It’s politics. It’s like some minority groups. Minorities have a loud voice that can be heard over shouting. They are creators of opinions. (SI-ST)*

#### 3.3.3. Perceptions with Regard to Future Changes

The Slovenian pork sector is strongly influenced by European trends and competition from abroad. The sector is unlikely to make any changes unless there is a clear requirement from abroad or a law banning castration. Although both major cooperatives are willing to prepare for a possible change, they are not proactively seeking any shift to the entire-male method or immunocastration. Their strategy is to wait and see what happens.

### 3.4. Germany

#### 3.4.1. The Structure of the Production/Market System and the Development of the Issue

From both the economic and political perspectives, Germany is regarded as highly influential in the economic activities of the EU. The Germany pig industry is the EU’s largest producer, consumer, and trader of pork [47]. German pig farming is based on the division of labor. More specifically, the majority of pigs are reared in farms that are specialized in either producing piglets or fattening pigs. Moreover, the production structures in pig farming vary greatly across the different regions of Germany. Pig farming is largely concentrated in the north-west, in the states of Lower Saxony and North Rhine-Westphalia. The farms in this region are predominantly of intermediate size, accounting for more than 60% of Germany’s total production [48]. In central and southern Germany, pig farms are relatively small [48]. As is the case with production, the German processing sector is characterized by diversity. It is important to note that the German processing sector is regarded as one of the most competitive in Europe, and it is generally efficient. The three leading processors—Tönnies, VION Germany, and Westfleisch—together account for 55% of the market [49].

Similar to the situation in the Netherlands, the focus of consumption in Germany is on bulk fresh meat, with 74% of all meat being sold in supermarkets [50]. In contrast to the Dutch market, however, Germany also has a strong market for regional and processed meat products (e.g., different kinds of sausages). Studies have shown that locally produced food is important to consumers, especially in the southern part of Germany.

With regard to the issue of piglet castration, the majority of piglets in Germany are surgically castrated [51]. In 2013, Germany outlawed the practice of surgical pig castration without anesthesia and offered a five-year transition period (until 2019). The transition period, however, was extended until 2021, to the outrage of animal-interest groups. For example, PETA, an animal rights group, filed a lawsuit with Germany’s Constitutional Court in November 2019. Public attention to the issue is increasing. The industry is actively searching for ways to cope with the societal pressure and remain competitive in the EU market. Due to Germany’s powerful position in the EU market, many producers in other EU member states are waiting to see what this country will do after 2021 [18], when the law is set for implementation.

In 2008, one year after the adoption of the Dutch “Noordwijk Declaration”, the German Farmers Association, the Association of the Meat Industry and the Association of German Retailers also signed a sectoral agreement to abandon the castration of piglets by 2017. The process of realizing the goal has been more of a struggle than it has been in the Netherlands. The greatest challenge involved with resolving the castration issue is thus that opinions are divided—not only amongst stakeholders from different groups, but also amongst stakeholders within the same group across regions—thereby resulting in endless discussions. As highlighted by an officer in one German state:

*This whole discussion is no longer based on facts. The fronts are just totally hardened and, somehow, one shoots at the other. These discussions go around and around. (D-GV)*

Interviewees identified several reasons underlying the slow process. First, the struggle with regard to the castration issue in Germany is related to its political structure and the coexistence of multiple pork-production systems in different states/regions. The Federal Republic of Germany has a federal constitution, and it consists of 16 partly sovereign constituent states. As noted by a manager from a slaughterhouse:

In Lower Saxony and North Rhine-Westphalia, boar fattening is the most widespread. This has to do with the marketers there, who took up the issue at a very early stage—together with us and some of our competitors. In the south, there is considerable resistance, due to the small structures and distinctive butcher marketing. Then there are also federal states, where there is obviously a discussion about the assessment of these animals in the slaughterhouse, as animals with a distinct sexual odor are confiscated and, depending on the interpretation of the responsible person on-site, there might be a higher or lower confiscation quota. If this quota exceeds a certain level, it is not possible to market them to these locations. There are major differences from circle to circle. (DE-SH)

Another reason underlying the slow pace of the process is that the fact that none of the existing alternatives are perfect in all technical, social, and economic respects has contributed to disagreements during stakeholder debates. More specifically, stakeholder debates concerning which method is the most appropriate tend to become infinite, as there are inevitably counterarguments to emphasize the disadvantages of any method (Table 1). The local-government officer and an NGO expert observed that some the opinions of some professionals are not entirely correct, thereby endangering progress in resolving the issue. The officer gave the following example of divided opinions among veterinarians:

*There are still many veterinarians who are opposed to immunocastration, for whatever reason, and who argue that local anesthesia is justifiable or that farmers should continue (castrating) as before. These veterinarians have thwarted the whole procedure. An opinion from a veterinarian is very dangerous if it is wrong, because it still comes from a veterinarian. (D-GV)*

Stakeholders can always cite results that are in favor of the positions that they have already adopted. In addition, new studies of inconsistent results provide fuel for new arguments. Debates focusing on identifying a single best method are inconclusive.

A third reason that progress has been slow is that the industry lacks collaboration and lacks a leadership role with regard to coordination. The action plan for resolving the issue is not clear, despite the frequent organization of stakeholder debates. In the absence of coordination, there is little room for negotiation, as people adopt fixed positions based on their own self-interest. Consequently, chain actors tend to take their own decisions to their own benefit. For example, large-size producers that have resources—including slaughterhouses (e.g., Tönnies and VION) and retailers that own processing units (e.g., Rewe and Edeka)—often invest in research and personnel to prepare themselves to accepting all methods, in case there is pressure from the market. The greatest uncertainty is thus experienced by small and medium-sized companies that are incapable of making large investments.

A fourth reason has to do with uncertainty regarding the use of anesthetics. The use of immunocastration and the raising of entire males both demands a fundamental change in the chain. Castration with anesthesia calls for the least adjustment and coordination in the supply chain, as the only change that is needed is at the level of the farm: farmers apply anesthetics when castrating pigs. In this regard, the industry—especially slaughterhouses/processors and retailers—is more in favor of accepting pigs that have been castrated by anesthetics, despite concerns expressed by farmers with regard to the safety of anesthetics. As observed by a representative from a farmers’ association:

*Each method has disadvantages. For example, isoflurane poses a major unknown risk with regard to the working safety of piglet producers. (D-FM)*

In addition to concerns about usage safety, several veterinarians and animal-interest groups have strongly criticized the use of anesthetics, as it is difficult to monitor its usage at the farm level. In Germany, the choice of anesthetics appears to be another major topic that adds complexity to the issue of castration.

#### 3.4.2. Stakeholder Engagement

In response to the identified challenges posed by the castration issue, experts are calling for the establishment of a platform/leadership institution to facilitate the coordination. The interviewees frequently cited examples from the Netherlands. They suggested two ways to implement a coordination platform. One involved introducing a certification system, such as the Beter Leven label in the Netherlands. Given that a certification system can help to segment the market, the development of new criteria for the evaluation of carcasses is the key to any labeling system. As noted by a manager from a leading slaughterhouse:

*The government’s animal-welfare label is too expensive, and there is a lot of chaos for the farmers, who are not aware of what to do. In the end, the farmer is the victim. In my opinion, stakeholders should replan the whole thing. As a slaughtering company with an animal-welfare program, we cannot market the whole pig. In the Netherlands, they are doing a better job with the Beter Leven programme. There, it is also possible to be economically efficient. (D-SH)*

The second way to implement a coordination platform is to introduce a payment scheme for differentiating pork from different methods in the slaughter line. The two possibilities are similar, in that criteria are needed in order to regulate the market. A manager from a German retailer explained how they had tried to influence the sourcing:

*I go out to the companies, and then we also develop our own catalogues of criteria. Thereafter, the farms will have to keep the animals [according to the criteria]. Everything related to the topic of castration is associated with different qualities, including test productions and sniffing the boar odor. This is why I have the coordination. We thus have influence with regard to the criteria. (D-RT)*

Farmers and slaughterhouses/processors are calling for more commitments and clearer statements from supermarkets with regard to their acceptance of alternatives. This is because the decisions of producers are likely to be affected by the question of whether and to what extent supermarkets will actually accept pork produced with alternative methods. To our knowledge, only two retailer chains, Rewe and Edeka, have taken action towards resolving the issue. Other retailers have stated that they accept all alternatives. According to interviewees from social pressure groups, the farmer association and a slaughterhouse, however, it is unclear whether retailers indeed accept all methods in practice. As a manager from a leading slaughterhouse pointed out,

*It is just political that they (supermarkets) say that they can accept everything. They always say publicly that they will accept the methods that consumers prefer. The supermarkets say, “We are not the last link in the chain” (D-SH)*

Stakeholders expect politicians to be involved in and properly coordinate the industrial debate. According to the interviewees, however, the coordinating role is not being fulfilled. At the time of writing this report, the Minister of Food and Agriculture had approved the “Ordinance on administering anesthesia with isoflurane in the castration of piglets by qualified persons” (FerkBetSachkV). The regulations allow farmers and other qualified individuals to apply general anesthesia with isoflurane. The government will allocate a total of EUR 20 million in 2020 to promote the purchase of anesthetics, thereby providing financial support to livestock farmers. Slaughterhouses/processors and retailers support this policy, reasoning that it would resolve the animal-welfare problem of castration and that “everybody will accept it” (DE-SH). In contrast, farmers—who will have to invest in inhalation agents and endure the potential risk of exposure to isoflurane (Long-term exposure may cause chronic or adverse health effects, including nausea, dizziness, fatigue, headache, irritability, reduced mental performance, liver and kidney disease, as well as possible reproductive effects such as sterility, infertility, miscarriages, and birth defects) —are suspicious of administering isoflurane for reasons of safety. Social pressure groups and some local governors have also criticized the Minister of Food and Agriculture or “directing a path for the industry”. The officer made this point by suggesting a hypothesis:

*If Mrs Klöckner (the Federal Minister of Food and Agriculture) had said that we are not investing this amount of EUR 20 million in isoflurane, but in immunocastration, we would now be on a completely different path. (D-GV)*

#### 3.4.3. Perceptions with Regard to Future Changes

The future of the castration issue is not entirely clear for farmers with regard to which method should be chosen and whether local anesthesia will be approved. In general, the experts were convinced that all three alternatives will coexist, with some increase in the possibilities for the production of immunocastrated pigs and entire males. Opinions differ with regard to estimating the market percentage of immunocastration and entire males. Some experts are convinced that immunocastrates may continue to form a niche for special products, while others argue that the market percentage for immunocastrates could reach as high as 30%. To prepare for an uncertain future, several large retailers and slaughterhouses have invested in preparing for all possible scenarios.

## 4. Discussion

This study aims to identify socioeconomic factors that inhibit European pork production chains from banning the painful castration of piglets. To do so, we address four questions, namely: how the castration issue has developed in four countries, why the development in these countries differ from each other, how the structure of the production system has influenced the stakeholder debate, and toward which direction stakeholders are moving. Results from a multiple case analysis highlight that the structure of the production and market of the pig sector is influential in shaping the development of the castration issue, motivating stakeholder debates in handling the issue, and affecting stakeholders’ positions towards different methods.

### 4.1. The Influence of the Development of the Castration Issue

Our multiple case study results display differences in issue development between the Netherlands, France, Slovenia, and Germany. In the Netherlands where the welfare problem of piglet castration was recognized earlier than in other European countries, production actors have worked together with NGOs to manage the issue. At the current stage, the Dutch industry has settled with entire-male production and animal interest groups have been satisfied with the solution. While in France and Germany where the public awareness about the castration issue is still growing, production actors are at a phase of debating about the right methods. In Slovenia production actors are less active in adjusting their way of production to either alternative method.

### 4.2. The Influence of the Production and Marketing System

An important factor contributing to the differences in the issue development and stakeholder debate is the variance of structure of production and market systems. Producers’ choices or preferences toward a certain alternative are largely dependent on their involvement in different types of production and market systems. For instance, the Dutch production system gears towards cost-efficiency [36,38]. As chain actors in this system are capable of coordinating costs and benefits and of marketing tainted carcasses, such as masking the tainted meat and making it into another product, converting to the entire-male solution can bring most economic benefits to the efficiency-oriented production chain. Comparably, also Cooperl, another example of efficiency-oriented production system in Brittany, France, opts for entire males. Another comparable example of cost-efficient system was found in Lower Saxony and North Rhine-Westphalia of Germany, likewise with a strong preference for entire males.

Compared to the Netherlands, France and Germany have more diverse production systems as they also produce regional specialties and other high-end products. In France, consumers have a relatively strong preference for meat quality, often with specific product preferences differing between regions. Thus, parallel to the cost-efficient system of Cooperl, more quality-oriented and more regionally based systems exist as well in France. In southern Germany where local production chains also generate value from their regional traditions farmers and slaughterhouses tend to be more conservative in making adjustments in their system. This explains their reluctance towards entire male and immunocastration solutions. Local chain actors are afraid of the possible negative reactions of consumers as the perceptions and taste of their products should be beyond any doubt. Farmers that are part of these regional chains therefore prefer to use anesthetics.

In Slovenia where the societal attention to the issue is small and where producers face intensive competition from abroad [28,45], large cooperatives prioritize the development of brands that emphasize the traditional value of the products to protect their domestic market rather than an animal welfare issue that consumers are not even aware of [13]. Therefore, the focus of the two dominant cooperatives is to strengthen their traditional brand image and reduce the production cost as much as possible. From this perspective, choosing entire male or immunocastration methods will not bring any benefits to their business. The cost of implementing the detection methods or using vaccines will increase costs and may affect the traditional value of their products [52]. Therefore, they too prefer castration with anesthesia applied by their own veterinarians. For local family farmers in Slovenia, all three methods are costly. Since the societal attention to the issue is small, family farmers do not face the same level of critics as those in the Netherlands, France, or Germany. Therefore, they are not interested in the discussion of the castration issue, accordingly they are not considering to switch voluntarily to any alternative. However, when experiencing high competition from abroad, family farms may have to change their practices, either well-prepared or not. Overall, in Slovenia, farmers or cooperatives will stay inactive for the issue.

### 4.3. The Direction of Stakeholder Debates in Different Production and Market Systems

The transition from castration to entire-male production in the Netherlands [36], Northern part of Germany [49], and Cooperl implies that production actors who belong to the efficiency-oriented system are driven by economic motive, stimulating chain actors to jointly implement the method of entire-male production. The entire-male solution is particularly complex as it requires efforts from several parties in the chain [53]. The benefit of the solution is that it reduces the costs (and therefore increases profits) for the efficiency-oriented production systems. While in France and Germany large vertically integrated companies took the lead in the debate (a German retailer in North/South and Cooperl, respectively), in the Netherlands the role was taken by a respected animal welfare organization (DSPA). These positive examples suggest that an efficient coordinating system plays a crucial role for implementing the entire-male solution.

Noticeably, the Dutch consensus on a single method is unlikely to prevail in other European countries because the Dutch system is mostly restricted to the national borders using the same language, legislation and institutions [16], and because it is by far the largest pork production system in the country, especially when compared to countries like France and Germany. In countries that inhabit different production systems of a significant size [23], stakeholders have a much greater difficulty determining which method is best. The same applies to the EU as a whole. As discussions for a single solution will always encounter disagreements, organizing a stakeholder debate will not be a “silver bullet”. The results of our study reflect how stakeholders see the issue evolving in the future. In general, most of them are positive about the possibility of solving the issue and are willing to abandon castration without anesthesia/analgesia. Their preferences for alternatives, as discussed above, largely depend on contextual factors such as the structure, scale, and cost and quality orientation of the production system.

Therefore, allowing the coexistence of different alternatives may be a way out for the castration issue. By doing so, the purpose of improving animal welfare is served. However, this solution may disturb the existing market flow in the European common market, if to compare with a simple and uniformed market where all piglets are castrated. However, this simple objective of having a uniform with a single method seems to ignore that the different alternatives come with benefits and costs that differ between pork production and market systems: Europe is rich in diversity including differences in efficiency, quality orientation, and regional orientation of the pork systems [23]. Harmonizing the configurations of pork systems is unlikely, if not impossible without severe economic consequences, because the different systems ultimately serve different consumer groups with different preferences. Allowing the different solutions to coexist may therefore be the fastest way to solve the castration issue and halt the unnecessary suffering of piglets. Legislation may then develop protocols on how to apply the different methods to minimize animal suffering.

## 5. Conclusions

In conclusion, the structure of the production and marketing system has a large influence on the way in which production actors make decisions. This is an important finding for the future progression of the castration issue. Some countries apply multiple systems with different orientations (e.g., cost-efficient, quality-oriented, regional/special products-oriented) and trade-offs between solutions. This study highlights the fact that differences between systems are apparently of greater importance than differences between countries, between stakeholder groups, or between various links in the supply chain. Given that the European single market hosts multiple systems, the implementation of a single solution would likely favor some systems at the expense of others. Therefore, an acceptable solution for the castration issue might be more easily identified if the issue was framed in terms of production systems rather than of national borders. In Europe, allowing all alternatives with protocols to coexist to satisfy the diverse market demands might be better for the European economy and for animal welfare.

## Figures and Tables

**Table 1:** Analysis of the pros and cons of available alternatives as compared with the standard surgical castration without any pain relief.

Alternative		Advantage	Disadvantage
SC	With anesthesia and/or analgesia	Reduced pain during SC	Extra costs (need authorized drugs and trained personnel) Maybe conflict with animal welfare
IC	Injecting vaccines	Better economic efficiency from prolonged fattening	Extra costs (need authorized drugs and safety training, detection at the slaughter line) Uncertain acceptability of consumers (and criticisms from chain actors)
EM	Slaughter before puberty	Reduced risk of boar taint No conflict with animal welfare	No guarantee of total elimination of boar taint Low economic efficiency
	Selection method (e.g., human nose detection in the slaughterhouses)	No conflict with animal welfare Better economic efficiency from prolonged fattening	No guarantee of total elimination of boar taint Extra costs (detection at the slaughter line) Lower meat quality

Simplified from: [2,3]. Note: * in comparison to the method of surgical castration without any pain relief.

**Table 2:** EU countries that have taken legislative actions regarding the practice of SC without pain relief.

Country	Year	Details
Norway	2002	Ban on surgical pig castration without analgesia and anesthesia
Netherlands	2009	A collective agreement of the Dutch food retail organizations
Switzerland	2010	Ban on surgical castration without anesthesia
Denmark	2009 2011	Ban on surgical castration without analgesia ^1^ (industry requirement since 2009, legal requirement since 2011)
Sweden	2016	Ban on surgical pig castration without analgesia and anesthesia
Germany France	2021 2021	Ban on surgical castration without anesthesia Ban on surgical castration without anesthesia

Adjusted and updated from: [17]. ^1^ The sequence may vary. According to Federation of Veterinarians of Europe, the combination of local or general anesthesia with analgesia is the best practice when SC is carried out.

**Table 3:** Population shares of male pigs raised as boars, immunocastrates, or barrows in Europe in 2017 [28,32], ranked according to size of pig population.

Country	Boars (%)	Immunocastrates (%)	Barrows (%)	Pig Population (×1000)
Netherlands	65	0	35	12,013
France	22	<0.1	78	11,835
Slovenia	1	0	99	288
Germany	20	<1	80	28,046

**Table 4:** Representative quotes from interviewees.

Country	n.	Interviewees Interest Groups	Description	Abbr.	Representative Quotes
NL	1	Farmer	The owner of a pig farm (size of 5000 fattening pigs and 600 sows)	NL-FM	“Our main goal is to avoid castration in a market-driven way. It means that almost all Dutch retailers ask for non-castrates.” “We work together in the pork chain, including pig farmer association, slaughterhouses, the Dutch ministry, and NGOs.”
	2	Slaughterhouse/processor	VION, the largest slaughterhouse in the Netherlands	NL-SH	“When you take a meta-view over it [the castration issue]. The impact is very limited. But the point is that every change creates resistance. To adapt your systems to non-castration needs some creativity.” “When you have one percent of the tainted boars, you have to process them in other way. We still can make meat products out of tainted carcasses. It’s possible, people know how to do it.” “I think raising entire male is the ultimate goal. It has all the benefits. For the other methods, you stay on castration and you have pain relief.”
	3	Retailer	The largest retailer in the Netherlands	NL-RT	“We were from the very beginning involved in introducing the “beter leven”. And we work closely with VION [a slaughterhouse]. Vion is contracting the farmers. We set criteria together and it is still developing”. “Most Dutch retailers agree on the criteria. The price increased a bit, but it is still a level playing field.”
	4	Breeder	Providing innovative genetic solutions for cost-efficient pig production.	NL-BR	“We, as breeders, always need to think from a future perspective. We have to think the future needs of our customers.” “Why should a farmer buy immunocastration if he does not get more payment for the extra investment? Though you do see people buying immunovaccines, but those are integrated producers.”
	5	Scientist	An experienced researcher	NL-ST	“The biggest challenge for the Dutch pork industry is the issue development in Germany. And at the same time, what makes it more complex is that in Denmark and in the Netherlands, each uses their own methods for pain relief. Denmark uses local anesthesia and in the Netherlands inhalation is carbon dioxide.”
	6	Animal protection	Dierenbescherming (the largest animal protection organisation in the Netherlands)	NL-NGO	“I think [raising entire males] is good for the image of the pig industry and pig farming. For farmers, they don’t have to castrate and enjoy a feed conversion benefit. It’s also economically interesting for them. ” “Why should we use immunocastration if we have a better solution [i.e., entire males]?” “We (different interest groups) don’t always agree with each other. We look for common ground and work on subjects.”
	7	Media	Pig progress	NL-ME	“I think what humans have been doing is trying to modify the pigs, to squeeze them into our production systems. And I think what needs to happen is the other way around: how can we find production systems that match the needs of the pigs and adjust our systems to how pigs are.” “I’m trying to be as balanced as possible. As a writer, I wouldn’t write anything enormously radical. I also I need to find a balanced way to express myself. So I may have these ideas but don’t necessarily always write about them.”
F	8	Farmer	INAPORC (a farmer association)	F-FM	“The quantity of the fat in meat from entire males or immunocastrates is not nearly the same compared to castrates. That’s why we farmers would like to have access to the anesthesia [to castrate pigs]. So the priority is to find a solution about anesthesia.” “When we are in discussion with the NGO, we say it’s not a problem for the farmers [to change the method]. If tomorrow we are asked to produce entire male, it’s not a problem to change. We’re happy, if we earn the same money.” “There are a lot of slaughterhouse that never want entire males. Because if they use entire males to produce the dry ham and dry sausages, the quality is a problem” “The only solution to improve the current situation is to find a machine to make the detection to be 100% accurate.”
	9	Cooperative	Cooperl	F-CO	“When we compare the three methods, uh, I would say entire male production have the most benefits.” “In Cooperl, we make decisions easily because we have a completepork chain.” “In our cooperative, we have a protocol to share the cost and benefits [among chain actors]. We share the cost and benefits added by raising and selling entire males. Everybody earns more money. It’s a win-win.”
	10	Pharmaceutical	Zoetis	F-PH	“We have some improvac customers, most of them started six or seven years ago and they’re still using it. But all of them, they’re selling their products directly, short chains.” “A good thing is if Germany stops castration, it is going to be easier to make things change [in Europe]. If Germany accept immunocastration, then Netherlands could also accept. So at the end, Germany could be a turning point.” “If there is a competitor, we would like to have a co-marketing with the competitor, at least would be more powerful. So I think it may be better for us to have a competitor in the market.”
	11	Veterinarian	Veterinarian	F-VET	“We agree with the position paper proposed by the Federation of Veterinarians of Europe (FVE)^1^ that entire male is better than immunocastration and immunocastration is and better than castration with anesthesia/analgesia.”
	12	Scientist—Slaughterhouse	IFIP (the French Pork and Pig Institute)	F-ST ^1^	“The biggest challenge for the issue is to detect the boar taint. That is, the knowledge part. And also to provide facilities to detect. As long as we can easily detect the boar taint, we can easily improve the farming techniques to reduce it.”
	13	Scientist—General	IFIP (the French Pork and Pig Institute)	F-ST ^2^	“We will never finish with the social challenge. If we solve this question, we will have other social challenge, so we’ll always have. So I think we should keep going on solving problems with technology. And hopefully, the social challenge will be achieved.” “The [carcasses from the] north [of France] is less fat, less boar taint. They are interested by the entire male because it will get better economic growth. The South is more for quality, fat, high quality products. So they would prefer a castrated pigs. Each one has its own existing markets. France has all methods, it is diverse.”
	14	Scientist-Animal welfare	IFIP (the French Pork and Pig Institute)	F-ST ^3^	“The problem for this issue is the industry (referring to slaughterhouses and processors) to have a reliable method, to be sure that all the boar tainted male are selected.” “Even though you have tainted detection in the Netherlands, in Germany and in Cooperl in France using human nose detection, but slaughterhouses in France don’t trust that. “
	15	Animal activist group	WELFARM	F-NGO	“We tried to convince MPs to propose. At the same time, we also put pressure on the governments. So [our task is] to put pressure on the government, to raise people awareness, to raise a signature, to have a petition.” “I think we can deal with this issue. If everybody decided to deal with this issue, we can.”
D	16	Farmer	Farmer Association	D-FM	“We need all legal methods [to exist], because each farmer has different needs and circumstances.” “A big challenge is that all legal methods will also be equally accepted so that farmers can market their animals for a fair price, regardless of which method they have chosen.” “The additional cost and work that will be there for the pig fattener is something that bothers the farmers. There is a shift in work from the producer to the fattener.”
	17	Slaughterhouse/processor ^1^	VION	D-SH ^1^	“We don’t sell whole pigs to one customer anymore. Some parts go to Japan, some go to Italy, they have different positions. That makes it complicated and also the selection in the slaughterhouses, they have to be separated, which smaller companies can’t fulfill.” “You need to look at the practical side. Think about all these processes at a smaller slaughter company, they can’t handle that huge selection problem. It costs a lot of money in logistics.” “The cooperation among stakeholders is often not satisfying. We need a social concern in this debate, but most don’t understand that yet. And the politics says we take a leading role in Germany, which is totally not true. Politics has failed.”
	18	Slaughterhouse/processor ^2^	Tonnies	D-SH ^2^	“Our suppliers (farmers) can use all allowed methods, because we have different requirements of the customers.” “We market the pigs in the form of many different cuts. With different customer requirements, this complicates internal logistics enormously. A comprehensive commitment to all methods from all industries would be beneficial for us.”
	19	Retailer ^1^	Anonymous—North Germany	D-RT ^1^	“There are big differences between countries and cities or between regions, sometimes as if they are islands. Even with ministries and federal ministries, they say something but the state ministries say different.” “With our company, our market sometimes has a completely different assortment in different regions. We are structured in seven regions. The retailers have committed themselves to their region and have said whether they want it.” “We work together with slaughterhouses. We have a common database and also see which farmers are currently up to the requirements.” “We have to be more prepared for our farmers to be able to implement methods. Otherwise we are losing the farmers, then we could not sell any more meat. That’s why we’re looking, which alternatives can we give with a clear indication? And what the farmers are able to do? So that they can implement it as quickly as possible and economically as well.”
	20	Retailer ^2^	Anonymous—South West Germany	D-RT ^2^	“In the past five years, we made some changes, partly the separation of the flow of goods for certain bulk buyers, or the additional work with the different methods, such as test production. With the coordination of the different methods.” “From an economic point of view, we actually have no benefits from any methods for our company. Economically this does not really bring us anything, but it is an improvement in animal welfare. The main goal of the company is to make progress and to have a direct influence where we want to achieve improvements in animal welfare and, of course, to avoid surgical interventions on animals.”
	21	Quality assurance system	Qualitäts Sicherung	D-QS	“The biggest challenge is that a national solo effort will place a heavy burden on German sows and piglet breeders because national solo efforts do not solve the problem. Germany imports a lot of piglets and pig meat but also of exports pigs to neighboring European countries, so it is the right way to regulate something at EU level and to find a regulation with the neighboring countries at a legal level so that the procedures are implemented in the same way in different countries from where pig meat or piglets and slaughter pigs are important, in order to create a fair level playing field.”
	22	State commissioner	Baden-Württemberg Commissioner	D-GV	“This whole discussion is no longer based on facts. The fronts are just totally hardened and somehow one shoots at the other. These discussions go round and round.”
	23	Animal protection ^1^	DTB	D-NGO ^1^	“The biggest challenges are actually the communication between the different industries and the acceptance of the different alternative methods that are available.”
	24	Animal protection ^2^	PROVIEH	D-NGO ^2^	“The biggest challenges in general are that not all alternatives are animal welfare friendly.” “A few years ago, we favored only entire males, because there is no need for surgery. But of course, years of practice and experience have shown that there are some farms that can do this very well, but it is not yet suitable for mass production. In addition, the boar meat is not universally applicable in the processing. The logical choice is therefore the immunocastration because it offers many advantages, for example the animals are calmer, every farm can implement it without the large management expenditure of the boar fattening. We reject the surgical castration generally because we have alternatives with which can be done without.”
SI	25	Cooperative ^1^	Ihan	SI-CO ^1^	“Now we don’t think a lot about immunocastration. From our knowledge, immunocastration is costly in the production.” “We have some kind of problems with junk market from other countries (referring to imports). They have competitive advantages on low prices. Since the last 3–5 years, Slovenian consumers wants to have Slovenian original meat. Consumers are willing to pay 5–10% more. That’s our focus now, to be original.” “Castration with anesthesia is not so expensive for us. We have experts for the surgery [in the company]. The cost is not a big difference between a few years ago and now.”
	26	Cooperative ^2^	Panvita	SI-CO ^2^	“We are following things and keep ourselves updated with the information to know how to react if and when there is really a ban. If there is ban, we will just consider split fattening, meaning females will be fattened to higher weight, and the entire males to lower weights. But of course it is a challenge, because it needs some changes in the organization of work and so on. We will adapt.”
	27	Breeder	Jata Emona	SI-BR	“We simply just think about the castration issue. We are new in this business as well, so we are not producing pigs. So we are also a bit not informed. We are not on the same regarding this problem as other countries.” “We think it’s not possible to produce without any castration. Without any castration, it’s not possible to reduce the smell.”
	28	Government	Minister of agriculture	SI-GV	“We have no information about this castration problem from producers. We are now working on our strategy on pig meat markets. We intend to identify the needs for the pig meat sector. We make also SWOT analysis about the sector. In any of SWOT section, there is no words about castration.”
	29	Scientist	KIS (Agricultural Institute of Slovenia)	SI-ST	“I think as a research institution, we should not recommend anything. Our role is to show the advantages and disadvantages, pros and cons. This is our role. It is up to them (referring to the production chains) to decide, what do they need, what do they produce, how to use.” “There was a time we published our result about different methods in a national-level journal. We were attacked by smaller farmers as if we were promoting IC or changes in the sector. Then after that, we decided not to go into the large public with the information anymore.”
Outside selected countries	30	NGO	Anonymous	NGO	“It’s very difficult to find a common ground. Stakeholders have very different positions, and to be completely honest with you, some of these positions are non-scientific. Therefore, it is sometimes difficult to have an informative discussion. Even in the face of new evidence, even if you show that the solutions are there, some stakeholders do not budge, for cultural and economic reasons.”
	31	EU Commission	Kai-Uwe Sprenger	GV	“The role for the European Commission is to ensure that the internal market functions. If, this is not yet the case, if member states establish national rules that disturb the internal market, then the European Commission comes into play, into the game. The member states can accept each other’s different rules, or we establish harmonized rules. How these harmonized rules look like, this is difficult to say right now. It depends on what kind of compromise member states can agree.”
	32	The Spanish Association of Pig Livestock Producers	Miguel Angel Higuera	General	“We (Spanish pork industry) have not pressures from our consumer regarding piglet castration because the majority our production is entire males (without castration). So what are we focused? We focus on producing the best quality meat. Also we can, an increase in the same time in the animal welfare by raising entire males. For that reason, as we are one of the biggest producers of EM, we have not any problem with consumers regarding piglet castration”
	33	Feed provider	Taintstop	Feed	“For Taintstop, the problem is that we have got a lot of research work and all of validation trials in not only lab circumstances, also the practice farms. But we find it difficult to find customers. Because the farmers will pay for it by themselves if he has an advantage for selling his pigs. If he doesn’t get the money back, he doesn’t buy it. It’s the system around the food chain that has to make choices.”
	34	European Veterinary Organisation	Federation of Veterinarians of Europe	VET	“We gave a position paper earlier. The aim is to go to entire male production. And if this is not possible, then the second method we support is immunocastration. And if that is not possible, then Analgesia and Anesthesia Around castration.” “As a veterinary association, we focus on trying to explain the science behind. While a couple of years ago, scientific arguments were important in this whole debate, I have the feeling that now it has become an immense political issue and scientific arguments are less important.” “I think we have to see this issue in a wider context, you cannot look only at pig castration. There’s so many other things going on in the pig sector, it’s better to take a holistic view and think, how are we farming pigs today? And is this the way we want to farm pigs in the future, or do we need to make changes to that?”

^1^ In 2001, FVE adopted its first position paper on pig castration [33]. In 2009, the 2001 FVE position paper was reviewed taking into account new scientific research, the market availability of alternatives and changing societal views.

## Appendix A

Interview Guide

Warming-up questions

1. Can you tell me about your professional background? How did you end up in this job?
2. What is your duty now in this job?

[Thanks for sharing the information with us. Now that we have understood your background, we are curious about your opinions regarding the piglet castration issue.]

A. Choosing one castration method over the others

1. From a more general perspective, what are the biggest challenges brought by the piglet castration issue for the German pork industry? (Ask the informant to clarify the challenges by different aspects/levels: economic, political, or social challenge. It could be about customer concerns/satisfaction, NGOs/media pressure, or the change of cultural norm.)
2. Compared to the situation of the pork industry five years ago, did this castration issue influence your business? If it did, in which way it affects your work? (Pay attention, if the response is a personal or a professional response) (To be more specific, have you made any changes in your business? What are the reasons? Standing now, what makes them hesitate or pause at this point?)
3. Since there are three available methods, how do you evaluate the economic benefits of each new method to your organization?
4. Besides the economic benefits, are there other advantages of the three available methods to your organization?
5. Each method has its downsides as well, what are the disadvantage of different methods to your organization? (Porter five forces —consumers, retailers, buyer, slaughterhouses, government, moral/ethics, economics, future policies, etc.)
6. Some academic research shows that consumers may actually accept the immunocastration method, do you agree with it?
7. Now that you’ve mentioned several advantages and disadvantages of each different method, what are the reasons that you believe one of them is better than the other two? (maybe personal goals, political issues, financial concerns, ethical concerns)
8. You suggest xx method is better than the other two, is your organization applying it? If yes, do you encounter any difficulties? (internal operation and external collaboration). If not, what are the adjustments you would need to make to use your preferred method, or other methods for that matter?

B. Working in the supply chain

Are there challenges you encounter in applying your preferred method because of the lack of consistency regarding castration?

1. Do you observe other stakeholders interested in the same method as you are? By stakeholders, we mean your competitors, your suppliers or customers, and NGOs or the media. (Open-question)
2. Do you observe other stakeholders interested in the other two methods? By stakeholders, we mean your competitors, your suppliers or customers, and NGOs or the media. (Open-question) Correspondingly, why do they prefer other methods?
3. Assuming that the supply chain is going to take the method you preferred, what are the adjustments the supply chain needs to make? (e.g., logistics, new practices of work flow)

[To solve the castration issue, it needs effort from not only your organization but also the entire supply chain. We are also curious about how the pressure of the issue is communicated and understood along the supply chain.]

4. When was the last time you and other stakeholders discussed about this issue? [continue the question with the specific how and where: how did the conversation take place (formal/informal? In big or small groups? One on one? Where do they happen, in conjunction with another meeting? Who was present? Who involved?)]
5. By organizing the various discussions, what are you hoping to accomplish? [The informant may answer: (1) claiming the position, follow-up questions: how do stakeholders state their position for the issue (e.g., no opinion, flexible if others change, unnegotiable); (2) solving a conflict, follow-up questions are: what are the conflicts really about, are they about morals? Money? Or change the old way by doing things in a new way? Who is taking the leading role to solve the conflict? Is there anyone missing from the conversation?; (3) collaborate together to reach an agreement, follow-up questions are: who are involved in the collaboration and what are the motives of collaboration; is there anyone missing from the conversation?; (4) innovating together, follow-up questions: what is the new method being discussed in the meeting? who are involved in the innovation? Who is going to benefit the most from the innovation?
6. How do you understand the various ways of communications (verbal, written, formal, or informal) you have engaged in. How do these communications affect your practices, your relationships with stakeholders, and interaction with them?
7. Did external stakeholders join the discussions in the past? (e.g., NGOs, government, pharmaceutical companies, etc.) If they did, do they put pressures in your discussion?
8. How do you think your preferred method will benefit the industry as a whole?

C. Developing/adjusting business strategy

[Now that we’ve discussed your interest of another method, we are wondering whether and how you’ve prepared your business in dealing with the castration-related challenges.]

1. Thinking of the pork industry next five years, based on your prediction, how will the castration issue be resolved?
2. Based on your prediction, how are you going to adjust your business to the future market? Does your choice of production method play into your market development strategy?
3. What is the ideal outcome for this issue? Please specify it in terms of animal welfare, for your own business, for the (sustainability of the) industry, for the government, for the EU, for consumers?

Closing

1. Is there anything else I should be aware of about the issue?
2. Have I missed anything?
3. Is there anything you want to add?
4. Is there anyone you think I should talk to?

## References

[1] I Furnols M.F., Aaslyng M., Backus G., Han J., Kuznetsova T., Panella-Riera N., Semenova A.A., Zhang Y., Oliver M. (2016). Russian and Chinese consumers’ acceptability of boar meat patties depending on their sensitivity to androstenone and skatole. Meat Sci..

[2] Bonneau M., Weiler U. (2019). Pros and Cons of Alternatives to Piglet Castration: Welfare, Boar Taint, and Other Meat Quality Traits. Animals.

[3] Čandek-Potokar M., Škrlep M., Zamaratskaia G. (2017). Immunocastration as Alternative to Surgical Castration in Pigs. Theriogenology.

[4] Kress K., Weiler U., Schmucker S., Čandek-Potokar M., Vrecl M., Fazarinc G., Škrlep M., Batorek-Lukač N., Stefanski V. (2019). Influence of Housing Conditions on Reliability of Immunocastration and Consequences for Growth Performance of Male Pigs. Animals.

[5] Gispert M., Oliver M., Àngels Velarde A., Suarez P., Pérez J., I Furnols M.F. (2010). Carcass and meat quality characteristics of immunocastrated male, surgically castrated male, entire male and female pigs. Meat Sci..

[6] Von Borell E., Baumgartner J., Giersing M., Jäggin N., Prunier A., Tuyttens F.A.M., Edwards S.A. (2009). Animal welfare implications of surgical castration and its alternatives in pigs. Anim..

[7] I Furnols M.F., Gispert M., Guerrero L., Velarde A., Tibau J., Soler J., Hortós M., García-Regueiro J., Pérez J., Suárez P. (2008). Consumers’ sensory acceptability of pork from immunocastrated male pigs. Meat Sci..

[8] Di Pasquale J., Nannoni E., Sardi L., Rubini G., Salvatore R., Bartoli L., Adinolfi F., Martelli G. (2019). Towards the aban-donment of surgical castration in pigs: How is immunocastration perceived by Italian consumers?. Animals.

[9] Crowe S., Cresswell K., Robertson A., Huby G., Avery A., Sheikh A. (2011). The case study approach. BMC Med. Res. Methodol..

[10] Doolin B. (1998). Information technology as disciplinary technology: Being critical in interpretive research on information systems. J. Inf. Technol..

[11] De Vries R.B., Gordijn B. (2009). Empirical ethics and its alleged meta-ethical fallacies. Bioeth..

[12] Yin R.K. (2017). Getting Started: How to Know Whether and When to Use the Case Study as a Research Method. Case study research and applications: Design and methods.

[13] Tomašević I., Bahelka I., Čítek J., Čandek-Potokar M., Djekić I., Getya A., Guerrero L., Ivanova S.G., Kušec G., Nakov D. (2020). Attitudes and Beliefs of Eastern European Consumers Towards Animal Welfare. Animals.

[14] Zyglidopoulos S.C. (2003). The Issue Life-Cycle: Implications for Reputation for Social Performance and Organizational Legitimacy. Corp. Reput. Rev..

[15] Greenwood R., Oliver C., Lawrence T.B., Meyer R.E., Greenwood R., Oliver C., Lawrence T.B., Meyer R.E. (2017). Social Movements and the Dynamics of Institutions and Organizations. By Marc Schneiberg and Michael Lounsbury. The SAGE Handbook of Organizational Institutionalism.

[16] Greenwood R., Raynard M., Kodeih F., Micelotta E.R., Lounsbury M. (2011). Institutional complexity and organizational re-sponses. Acad. Manag. Ann..

[17] De Briyne N., Berg C., Blaha T., Temple D. (2016). Pig castration: Will the EU manage to ban pig castration by 2018?. Porc. Heal. Manag..

[18] Lin-Schilstra L., Ingenbleek P. A complex ball game: Piglet castration as a dynamic and complex social issue in the EU. J. Agric. Environ..

[19] Aggarwal R., Erel I., Starks L.T. (2014). Influence of Public Opinion on Investor Voting and Proxy Advisors. Fish. Coll. Bus. Work. Pap. J..

[20] Wartick S.L., Mahon J.F. (1994). Toward a substantive definition of the corporate issue construct: A review and synthesis of the literature. Bus. Soc..

[21] Storbacka K., Nenonen S. (2011). Markets as configurations. Eur. J. Mark..

[22] Krippner G., Granovetter M., Block F., Biggart N., Beamish T., Hsing Y., Hart G., Arrighi G., Mendell M., Hall J. (2004). Polanyi Symposium: A conversation on embeddedness. Socio Econ. Rev..

[23] Trienekens J., Petersen B., Wognum N., Brinkmann D., Plà-Aragonés L.M. (2009). Introduction to the European pork chain. European Pork Chains: Diversity and Quality Challenges in Consumer-Oriented Production and Distribution.

[24] Sommerfeldt E.J., Yang A. (2017). Relationship networks as strategic issues management: An issue-stage framework of social movement organization network strategies. Public Relat. Rev..

[25] Golob U., Podnar K. (2014). Critical points of CSR-related stakeholder dialogue in practice. Bus. Ethic-A Eur. Rev..

[26] Goodstein J.D., Wicks A.C. (2007). Corporate and Stakeholder Responsibility: Making Business Ethics A Two-Way Conversation. Bus. Ethic-Q..

[27] Kaptein M., Van Tulder R. (2003). Toward Effective Stakeholder Dialogue. Bus. Soc. Rev..

[28] Backus G., Higuera M., Juul N., Nalon E., De Briyne N. Second Progress Report 2015–2017 on the European Declaration on Alternatives to Surgical Castration of Pigs; Expert Group on ending surgical castration of pigs. Brussels, May 2018. https://www.boarsontheway.com/wp-content/uploads/2018/08/Second-progress-report-2015-2017-final-1.pdf.

[29] Volker S. Sustainability in Pork Production with Immunocastration. https://era-susan.eu/content/susi.

[30] Bleich E., Pekkanen R. (2013). How to Report Interview Data.

[31] Corbin J., Strauss A. (2008). Basics of Qualitative Research (3rd ed.): Techniques and Procedures for Developing Grounded Theory.

[32] Kress K., Millet S., Labussière É., Weiler U., Stefanski V. (2019). Sustainability of pork production with immunocastration in Europe. Sustainability.

[33] Federation of Veterinarians of Europe Pig castration—FVE position paper. https://fve.org/cms/wp-content/uploads/fve_09_040_castration_pigs_2009.pdf.

[34] Workman D. Pork Exports by Country. http://www.worldstopexports.com/pork-exports-by-country/.

[35] Press T.A. Sales of Flowers, Pork Push Dutch Farm Exports to New Record. https://abcnews.go.com/Business/wireStory/sales-flowers-pork-push-dutch-farm-exports-record-68349017.

[36] Kemp B., Soede N., den Hartog L. Pig production in The Netherlands: Analyses and trends. Proceedings of the 2011 Manitoba Swine Seminar.

[37] Ingenbleek P.T., Harvey D., Ilieski V., Immink V.M., De Roest K., Schmid O. (2013). The European Market for Animal-Friendly Products in a Societal Context. Animals.

[38] Pinckaers M. (2019). The Dutch Food Retail. Report 2019.

[39] Mahan A.T. (2017). The Present Predominance of Germany in Europe—Its Foundations and Tendencies. The Interest of America in International Conditions.

[40] France, third largest pig producer in Europe. https://www.rotecna.com/en/blog/france-third-largest-pig-producer-in-europe/.

[41] Marque P., Rabade T., Fortio R. (2014). Pig Farming in the European Union: Considerable Variations from One Member State to Another.

[42] Derksen B. Off the beaten path farms: France’s Perfect Pig Company. https://thepigsite.com/articles/off-the-beaten-path-farms-frances-perfect-pig-company.

[43] Weinrich R. (2018). Cross-Cultural Comparison between German, French and Dutch Consumer Preferences for Meat Substitutes. Sustainability.

[44] Hocquette J.F., Jacquet A., Giraud G., Legrand I., Sans P., Mainsant P., Verbeke W. (2013). Quality of Food Products and Consumer Attitudes in France. Energy and Protein Metabolism and Nutrition.

[45] Esselink H. (2009). Agricultural Production Chains in Slovenia.

[46] Augère-Granier M.-L. (2020). The EU Pig Meat Sector. https://www.europarl.europa.eu/RegData/etudes/BRIE/2020/652044/EPRS_BRI.

[47] Wilkins B. Fall in German Pork Consumption. https://ahdb.org.uk/news/fall-in-german-pork-consumption.

[48] McLay A. Germany a Powerhouse on Pig Production. https://www.foodnavigator.com/Article/2011/12/07/Germany-a-powerhouse-on-pig-production.

[49] Bittlmayer H. (2019). Local Characteristics of Pig Production in Germany and Bavaria.

[50] (2007). General Description of Pork Chains in Germany.

[51] Debate on piglet castration. https://www.bmel.de/EN/topics/animals/animal-welfare/debate-piglet-castration.html.

[52] Bonneau M. (2010). PIGCAS: Attitudes, practises and state of the art regarding piglet castration in Europe. Proceedings of Workshop “Castration of piglets”.

[53] Borrisser-Pairó F., Kallas Z., Panella-Riera N., Avena M., Ibáñez M., Olivares A., Gil Roig J.M., Oliver M.A. (2016). Towards entire male pigs in Europe: A perspective from the Spanish supply chain. Res. Veter. Sci..

